# Preparation and Characterization of Rifampin Loaded Mesoporous Silica Nanoparticles as a Potential System for Pulmonary Drug Delivery

**Published:** 2015

**Authors:** Meysam Mohseni, Kambiz Gilani, Seyed Alireza Mortazavi

**Affiliations:** a *Department of Pharmaceutics, School of Pharmacy, Shahid Beheshti University of Medical Sciences, Tehran, Iran. *; b *Department of Pharmaceutics, School of Pharmacy, Tehran University of Medical Sciences, Tehran, Iran. *; c *Pharmaceutical Sciences Research Center, Shahid Beheshti University of Medical Sciences, Tehran, Iran.*

**Keywords:** Mesoporous silica nanoparticles, Rifampin, Drug delivery, Drug-loading, Drug release

## Abstract

The goal of this research is to determine the feasibility of loading rifampin into mesoporous silica nanoparticles. Rifampin was selected as a model lipophilic molecule since it is a well-documented and much used anti tuberculosis drug. The mesoporous silica nanoparticles were prepared by using tetraethyl ortho silicate and cetyltrimethyl ammonium bromide (as surfactant). The prepared nanoparticles were characterized in terms of their particle size measurement and porosimetry. The results showed that the particle size is 218 ± 46 nm (mean ± SD) and surface area is 816 m^2^g^-1^. In order to load rifampin within the mesopores, adsorption experiments using three different solvents (methanol, water and dimethyl sulfoxide) were carried out. The loading procedure resulted in a significant improvement in the amount of rifampin loaded into mesoporous silica nanoparticles and methanol was found to be a suitable solvent, providing a drug entrapment efficiency of 52 %. Rifampin loaded nanoparticles underwent different *in-vitro* tests including, SEM and drug release. The *in-vitro* drug release was investigated using buffer phosphate (pH=7.4). Regarding the drug release study, a biphasic pattern of release was observed. The drug-loaded mesoporous silica nanoparticles were capable of releasing 95% of their drug content after 24 h, following a faster release in the first four hours. The prepared rifampin loaded nanoparticles seem to have potential for use as a pulmonary drug delivery.

## Introduction

Since drug-loaded particles are suitable for controlled release and drug targeting, they have been the focus of research in drug delivery systems (1). Developments in encapsulation technology have allowed the preparation of a large range of submicron-sized drug-loaded particles. These nanoparticles may have widespread potential as drug carriers due to the presence of an organic shell or to their organization (colloidal systems, liposomes, microemulsion, *etc*.) (-). Among these drug delivery systems, inorganic porous materials are emerging as a new category of host/guest systems. Due to some interesting features such as their biological stability and their drug-releasing properties (6), there is a significant and increasing interest in these potential carriers. Several porous minerals have been used including synthetic zeolithe, silica xerogel material and porous ceramic (-). MCM 41 is a typical mesoporous templated silica which has been largely investigated. This material presents nanosized pores that allow the inclusion of drug into the pores. In the past decade, mesoporous silica microparticles (MSMs) have found widespread application as controlled drug delivery systems (DDS) (9). Mesoporous silica nanoparticles (MSN) offer several attractive features, such as a large surface area, easily modified pore size and volume, as well as being chemically inert and allowing easier functionalization of their surface (-). All these features allow better control of drug loading and release. Administration of MSNs can take place through parenteral and oral route. One of their main advantages is the ability to increase the solubility of poorly water soluble drugs, while they can also be used for hydrophilic active agents. Thus, high drug loading can be achieved with loading capacities normally varying from 10 to 34% (20) or up to 60% in extreme cases (21). They have been also used for controlled release and drug targeting, providing sustained release for 16 h (22).

 Among the various drug delivery systems considered for pulmonary application, nanoparticles demonstrate several advantages for the treatment of respiratory diseases, like prolonged drug release, cell specific targeted drug delivery or modified biological distribution of drugs, both at the cellular and organ level (23). 

There has always been a concern for silica nanoparticles toxicity, but many studies have shown that this concern is undervalued. For example one study on early life stage of Zebrafish has shown that silica nanoparticles and/or aggregates mainly accumulate on the chorion of embryos and exhibit no overt emryotoxicity (24). Another study has shown that silica nanoparticles do not reduce glutathione level nor generating ROS in mouse keratinocytes (25). Yet another study has shown that single and repeated doses in intravenously exposed mouse cause no death (26).

Rifampin is an antibiotic against *Mycobacterium Tuberculosis, *which is widely used for the treatment of tuberculosis. The aim of this study is to determine the loading capacity of mesoporous silica nanoparticles and to characterize the drug-loaded particles. For this purpose, we synthesized a MSN with a distribution of pore sizes in the range from 1 to 3 nm. Rifampin, was selected as a model drug for its low solubility in water and its molecular size. The latter is suitable for its incorporation within the pores of MSN. After the synthesis of MSN, adsorption of rifampin on the channels surface of MSN was carried out using various solvents, as the interactions between the solute and the mineral surface depends on the solvent properties. Successive impregnations of the MSNs have been performed with a solution of rifampin in methanol. In addition, release behavior of rifampin loaded MSNs has been studied.

## Experimental

***Materials***

Rifampin was obtained from Alhavi pharmaceutical company, Iran. Tetra ethyl ortho silicate (TEOS), cetyltrimethyl- ammonium bromide (CTAB), dimethyl sulfoxide and phosphoric acid were obtained from Sigma (Germany). Triethanolamine, hydrochloric acid, potassium chloride, sodium chloride, methanol and sodium hydrogen phosphate (dibasic) were obtained from Merck (Germany). All chemicals were used as received.

***Synthesis of MSN***

The mesoporous silica nanoparticles were prepared using a general method where TEOS was added into an aqueous solution containing CTAB, ethanol, and additives such as inorganic salts, DEA or TEA. The method used in this study was as follows: 6.4 mL of water (0.36 mol), 0.9 g of ethanol (0.015 mol), 0.28 g of CTAB (0.786 mmol) and 0.02 g of DEA (0.19 mmol) were mixed and stirred in a water bath at 40 ˚C for 30 min. Then 0.73 mL of TEOS (3.25 mmol) was added into the mixture dropwise within 2 min under stirring. The solution turned white gradually. A further 2 h stirring was necessary. After that, the solution was cooled to room temperature. The white powder was centrifuged and washed with distilled water and ethanol. The surfactant (CTAB) was extracted by refluxing the obtained mesoporous materials (1 g) at 80 °C with 60 mL ethanol and addition of a small amount of concentrated HCl. The final product was obtained by centrifugation and washed with ethanol for several times (27).

***Loading procedure***

The passive method was used to load MSN with rifampin. Three different solvents were used for loading, depending on the polarity index. 2 mg of MSN was added to 2 mL of a 2 mg/mL rifampin solution. Afterward the suspensions were brought to equilibrium under gentle stirring for 24 h. The loading procedure was carried out by using successively dimethyl sulfoxide (DMSO), methanol and water as solvent. Apart from the effect of solvent, the influence of temperature and time on the loading procedure was investigated.

***Characterization of MSNs***

***SEM***

The morphology of the prepared samples was characterized using a scanning electron microscopy (SEM). The samples were attached to aluminum stubs with double side adhesive carbon tape then gold coated and examined using a scanning electron microscope (SEM, LEO 1455VP, Cambridge, U.K.).

***Porosimetry***

The pore characteristics of the mesoporous silica nanoparticles were studied by determining the nitrogen adsorption using a surface area and pore size analyzer (BELsorp-mini II, Japan) at −196 °C. MSNs were degassed at 100 °C for 3 h under argon gas flow before analysis, while the drug-loaded samples were degassed at 40 °C for 12 h. The pore characteristics were determined according to the Brunauer–Emmett–Teller (BET) and Barrett–Joyner–Halenda (BJH) procedures from the adsorption sections of the isotherms.

***Particle size measurements***

The particle size of the MSNs was measured with Malvern Zetasizer Nano ZS (Malvern, UK). The analysis was performed at a temperature of 25 °C, using samples appropriately diluted with filtrated and double distilled water in order to avoid multi scattering events.

***XRD***

XRD patterns of the samples were collected using an X-ray diffractometer (Philips X’pert, Netherlands) equipped with a liquid nitrogen-cooled germanium solid-state detector and Cu Kα radiation over a range of 0.8–10° 2θ.

***Determination of entrapment efficiency***

After the loading procedure, the suspensions were centrifuged at 14000 rpm for 20 min (Centrifuge 5418, Eppendorf AG) and rifampin that remained in the supernatant phase was determined using HPLC analysis with UV detection at 254 nm. Rifampin was analyzed by a Knauer HPLC system consisting of a 1000 pump and a 2500 UV–VIS detector (Germany). Analysis was carried out on a Nucleodur C8 column (150×4.6 mm, 5 μm). The mobile phase consisted of a 66:34 (%v/v) mixture of phosphate buffer and acetonitrile, the flow rate was 1.5 ml/min and the detection wavelength was 254 nm. The injection volume was 10 μL. 

Entrapment efficiency (%) = (weight of drug in nanoparticles /weight of drug fed initially) ×100.

***In-vitro drug release***

The *in-vitro* drug release study was carried out using a dialysis bag (cellulose membrane ,MW cut-off 12,400, Sigma–Aldrich), which (1) allows free diffusion of the drug molecules into the release medium, while at the same time (2) completely separates the nanoparticles from the release medium. About 10 mg of the drug-loaded nanoparticles were suspended in 2 mL of phosphate buffer solution (pH 6.8) inside a dialysis bag. The pH of the buffer solution was adjusted to 7.4. The dialysis bag was then placed in 38 mL of the buffer solution (sink condition) at 25 °C under magnetic stirring. At successive time intervals, aliquots (2 mL) of the release medium was collected and replaced with a fresh buffer solution. The collected sample was then analyzed using HPLC. The *in-vitro* drug release was carried out for 24 h. Each experiment was conducted in triplicate.

## Results and Discussion

***MSNs characterization***

**Figure 1:**
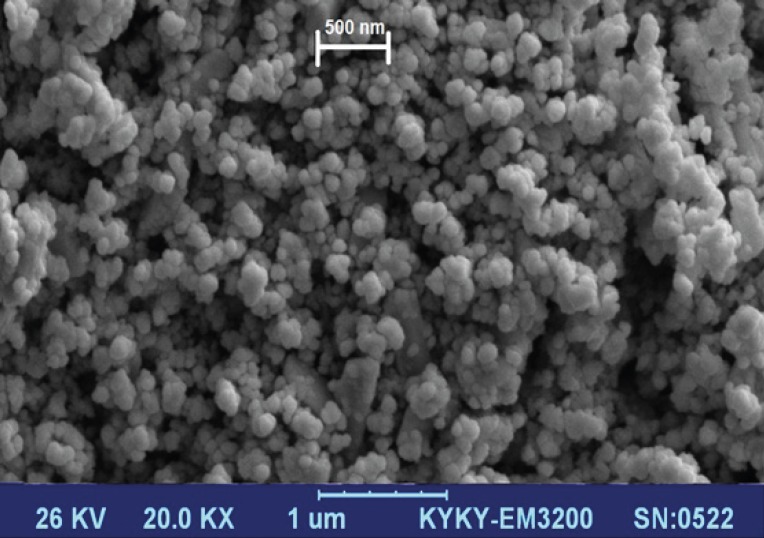
SEM of the synthesized mesoporous silica nanoparticles

Figure 1 displays scanning electron microscopy observations of drug-free MSN particles. Silica nanoparticles were observed as small spherical particles with a size of about 200 nm.

**Figure 2:**
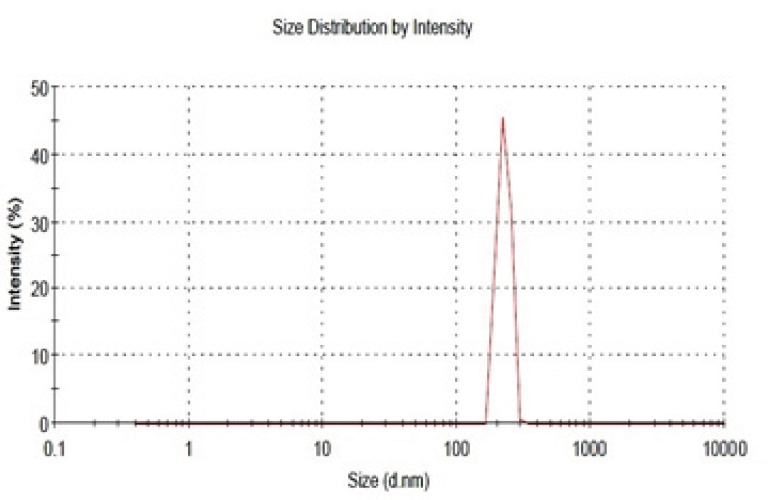
Size distribution of the prepared drug-free mesoporous silica nanoparticles

As mentioned, the particle size was determined with Zetasizer. The result obtained has been shown in Figure 2. As can be seen, the logarithmic particle size distribution is normal and the Z- average particle diameter is 218 ± 46 nm (n=3, mean±SD). Polydispersity index (PDI) and D_90_ are 0.2 and 290 nm, respectively. The size and sharpness of the peak indicates that the MSNs are suitable for preparation of nano-aggregates as a potential pulmonary drug delivery system. 

**Figure 3.**
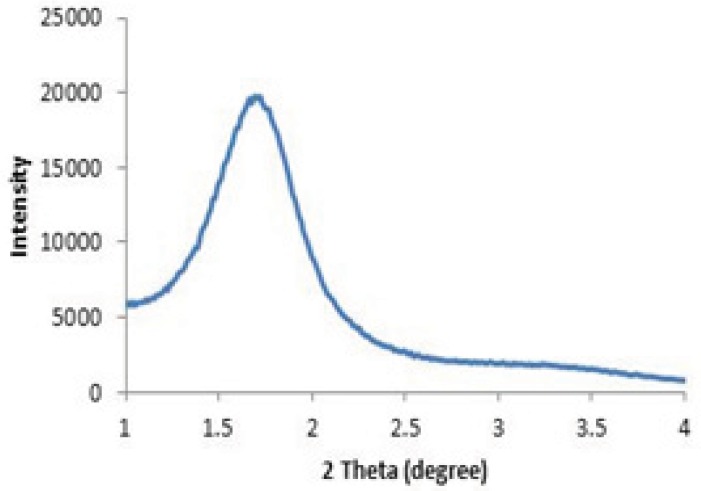
Typical XRD pattern of the prepared mesoporous silica nanoparticles

**Figure 4:**
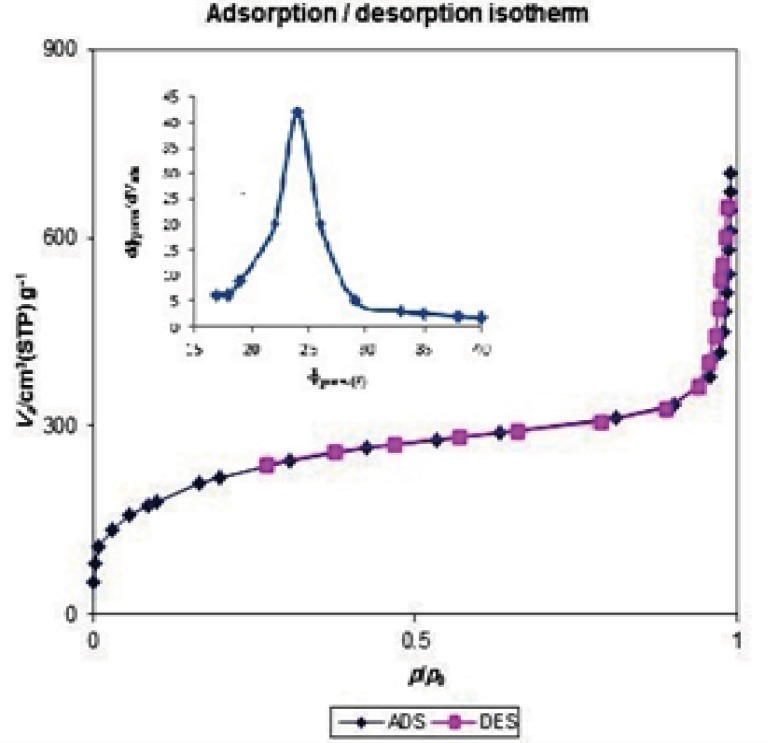
Nitrogen adsorption/desorption isotherm of prepared mesoporous silica nanoparticles.

The structural properties of MSNs used in this study have been determined by powder X-ray diffraction (Figure 3) and nitrogen adsorption (Figure 4).

As shown in Figure 3, a strong peak appears at a 2θ angle in the range of 2.2-2.4°. This diffraction peak reveals a regular periodic variation of the electron density due to the long range ordering of the pores in the mesoporous silica nanoparticles (28).

The N_2_ adsorption/desorption isotherm, shown in Figure 4 could be classified as type IV isotherm with a hysteresis loop, according to the IUPAC nomenclature (29). The observed hysteresis loop at a high relative pressure (about 0.9) can be attributed to inter-particle porosity, a feature that is often found for similarly prepared silica mesoporous materials (30, 31). The specific surface area of the synthesized MSNs material is 816 m^2^g^-1^, with a measured mesoporous volume of 1.0679 cm^3^g^-1^ and a narrow distribution for the pore size centered at 2.4 nm. The pore diameter was determined with the BJH method, based on the adsorption branch data. Therefore, all these data indicate that the MSNs used for this investigation exhibit a well ordered mesoporous structure.

***Drug Loading***

**Figure 5:**
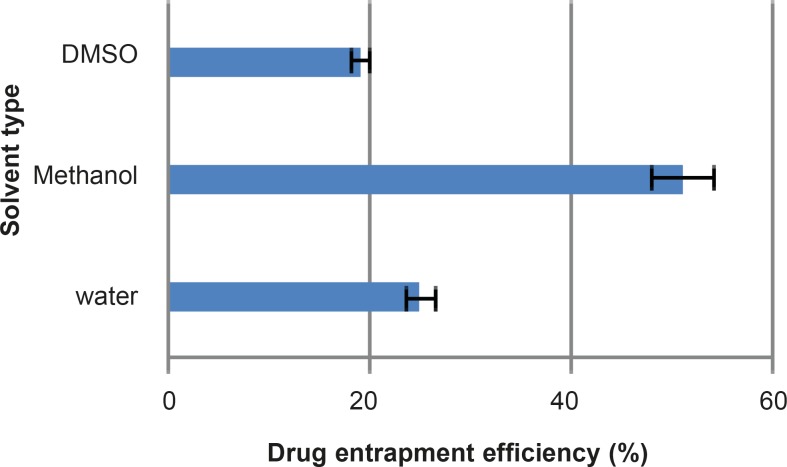
Rifampin entrapment efficiency (%) of three different solvents within MSNs at 25°C (n=3, mean±SD).

One of the main objectives of the current study was to prepare MSNs with an increased specific surface area and pore volume, in order to achieve high loading of drug molecules. The chosen mesoporous silica nanoparticles presented a high surface area and good pore volume, prior to the loading studies. Passive loading was chosen as the preferred method to load the active drug molecules and to increase the loading efficiency. Among the influential features of the loading process is the polarity of the organic solvent (32). The chosen solvents included water, methanol and dimethyl sulfoxide with polarity indices of 10.1, 5.1 and 3.1, respectively. The results obtained from the loading procedure are shown in Figure 5.

In some cases low polar solvents hamper the drug loading and essentially the whole process depends on the solvent properties. In this study dimethyl sulfoxide had minimum entrapment efficiency due to its low polarity and this could hinder rifampin molecules from loading into the nanoparticles. On the other hand, water with a high polarity index also had low entrapment efficiency which suggests an interaction between water and silica nanoparticles, preventing or limiting the loading procedure. Methanol had the highest entrapment efficiency among the three solvents studied. Hence, the influence of time and temperature on the loading procedure was investigated with methanol.

**Figure 6:**
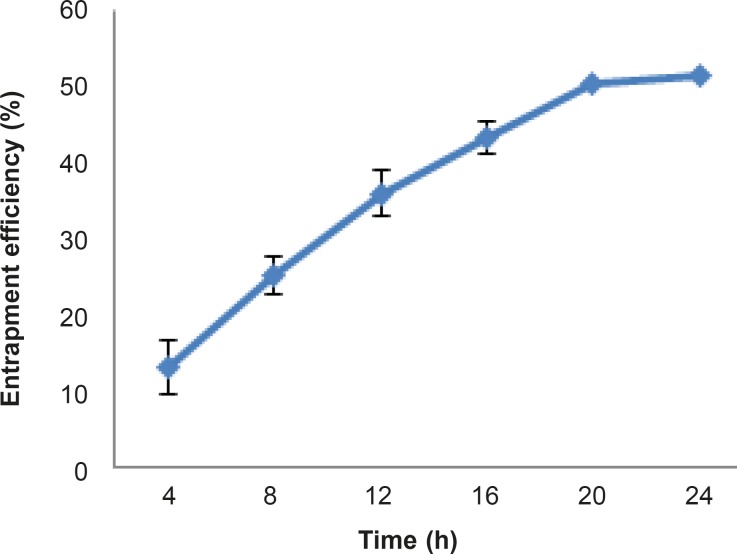
Entrapment efficiency (%) of rifampin within MSNs at 25 °C after different time intervals (n=3, mean ± SD).

In order to study the effect of temperature on the loading procedure, every four hours the loading was measured. The results have been shown in     Figure 6 . As can be seen, the entrapment efficiency increased with time until achieved a plateau between 20 and 24 hours after the start of the study.

In order to study the effect of temperature on the loading procedure, three different temperatures including 4 °C, 25 °C and 45 °C was chosen. The results show that entrapment efficiency at 4 °C and 25 °C were 25% and 51%, respectively. Lower diffusion coefficient of the drug at lower temperatures may explain this result. At 45 °C degradation of rifampin is fast, so the result of loading procedure at this temperature was not valid.

***In-vitro drug release***

Dissolution profile of rifampin loaded MSNs was investigated in phosphate buffer as the test medium. Accurately weighed amounts of the prepared sample was used under sink conditions (C < 0.2Cs). 

**Figure 7:**
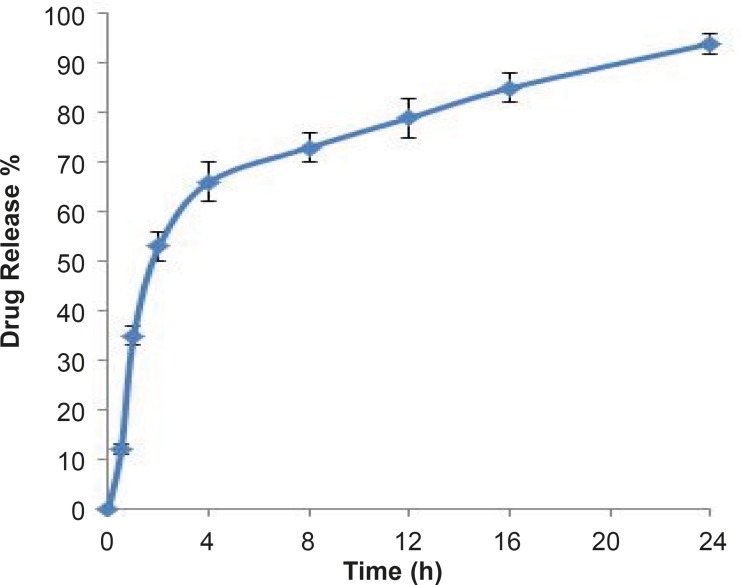
Profile of rifampin released from MSNs in pH 7.4 phosphate buffer medium at 25 °C (n=3, mean±SD).

Interestingly, as shown in Figure 7 a prolonged release pattern was observed for rifampin, following a faster release in the first four hours in which about 60 percent of drug was released. Overall, 95% drug release was achieved after 24 hours. The biphasic release can provide a high concentration of rifampin at first and then a slow release of drug could maintain the concentration of rifampin at a therapeutic level.

## Conclusion

The results obtained show that rifampin could be efficiently loaded into mesoporous silica nanoparticles. The loading extent is influenced by the loading procedure. Parameters such as the type of solvent, time and temperature are important. Successive impregnations of the MSNs within a solution of rifampin in methanol resulted in a significant improvement in the amount of drug molecules entrapped. For pulmonary drug delivery this rifampin loaded nanoparticles should be converted into nanoaggregate in order to deposit them within the lung.

Drug release studies showed a prolonged and complete release of rifampin molecules included within mesopores, principally by a diffusion phenomenon. Hence, it seems that association of a nanostructured mineral to the molecular state of the drug presents a great interest for pharmaceutical applications, as it allows control over the kinetics of drug delivery, especially for lipophilic drugs. 
